# Miniaturized FDDA and CMOS Based Potentiostat for Bio-Applications

**DOI:** 10.3390/s17040810

**Published:** 2017-04-10

**Authors:** Elnaz Ghodsevali, Samuel Morneau-Gamache, Jessy Mathault, Hamza Landari, Élodie Boisselier, Mounir Boukadoum, Benoit Gosselin, Amine Miled

**Affiliations:** 1 LABioTRON Bioeng. Research Laboratory, ECE Dept. Université Laval, Québec City, QC G1V 0A6, Canada; elnaz.ghodsevali.1@ulaval.ca (E.G.); samuel.morneau-gamache.1@ulaval.ca (S.M.-G.); jessy.mathault.1@ulaval.ca (J.M.); hamza.landari.1@ulaval.ca (H.L.); 2 Ophthalmology Department, Faculty of Medicine, Université Laval, Québec City, QC G1V 0A6, Canada; Elodie.Boisselier@fmed.ulaval.ca; 3 CoFaMic, Université du Québec à Montréal, Montreal, QC H2L 2C4, Canada; boukadoum.mounir@uqam.ca; 4 Biomedical Microsystems Laboratory, ECE Dept. Université Laval, Québec City, QC G1V 0A6, Canada; benoit.gosselin@gel.ulaval.ca

**Keywords:** electrochemical sensor, electronics, integration

## Abstract

A novel fully differential difference CMOS potentiostat suitable for neurotransmitter sensing is presented. The described architecture relies on a fully differential difference amplifier (FDDA) circuit to detect a wide range of reduction-oxidation currents, while exhibiting low-power consumption and low-noise operation. This is made possible thanks to the fully differential feature of the FDDA, which allows to increase the source voltage swing without the need for additional dedicated circuitry. The FDDA also reduces the number of amplifiers and passive elements in the potentiostat design, which lowers the overall power consumption and noise. The proposed potentiostat was fabricated in 0.18 µm CMOS, with 1.8 V supply voltage. The device achieved 5 µA sensitivity and 0.99 linearity. The input-referred noise was 6.9 µV${}_{\mathrm{rms}}$ and the flicker noise was negligible. The total power consumption was under 55 µW. The complete system was assembled on a 20 mm × 20 mm platform that includes the potentiostat chip, the electrode terminals and an instrumentation amplifier for redox current buffering, once converted to a voltage by a series resistor. the chip dimensions were 1 mm × 0.5 mm and the other PCB components were off-chip resistors, capacitors and amplifiers for data acquisition. The system was successfully tested with ferricyanide, a stable electroactive compound, and validated with dopamine, a popular neurotransmitter.

## 1. Introduction

Understanding electrical signal transmission in the brain and the neurotransmitter (NT) exchanges that give rise to it plays an important role in the study of human behavior and the development of therapies for diseases such as Alzheimer and Parkinson [1,2,3]. A brain neuron typically communicates with another neuron by using an action potential (AP) that originates in its axon hillock and propagates down to its synaptic terminals. Then, the AP triggers the release of neurotransmitters that diffuse across the synaptic gap to another neuron, at which point a postsynaptic potential is created [4]. This process continues until the transfer of information is completed. When brain dysfunctions occur, such as in Parkinson and Alzheimer diseases, it has been established that NTs like dopamine, glutamate and serotonin are involved [5].

Different techniques had been proposed to detect NTs, of which chemiluminescent imaging, liquid chromatography, positron emission tomography (PET) and single photon emission computerized tomography (SPECT) [6,7]. These techniques are expensive and use complex equipment. Consequently, electrochemical biosensors have received much interest to do the task at a lower cost, and with high accuracy, selectivity and sensitivity [8,9,10,11].

One of the most widely used transducer principles in electrochemical sensors is amperometry, which measures the redox current resulting from the electron exchange between a chemical solution and an electrode surface, hence providing a means to measure the concentration of a desired analyte in the solution. The potentiostat is one such circuit. It operates by applying and maintaining a constant voltage drop across two electrodes and an analyte in solution, and measuring the electrical current from the ensuing chemical reactions. When a bidirectional voltage ramp is used, the sensing technique is called cyclic voltammetry (CV) [12,13,14].

**Figure 1:**
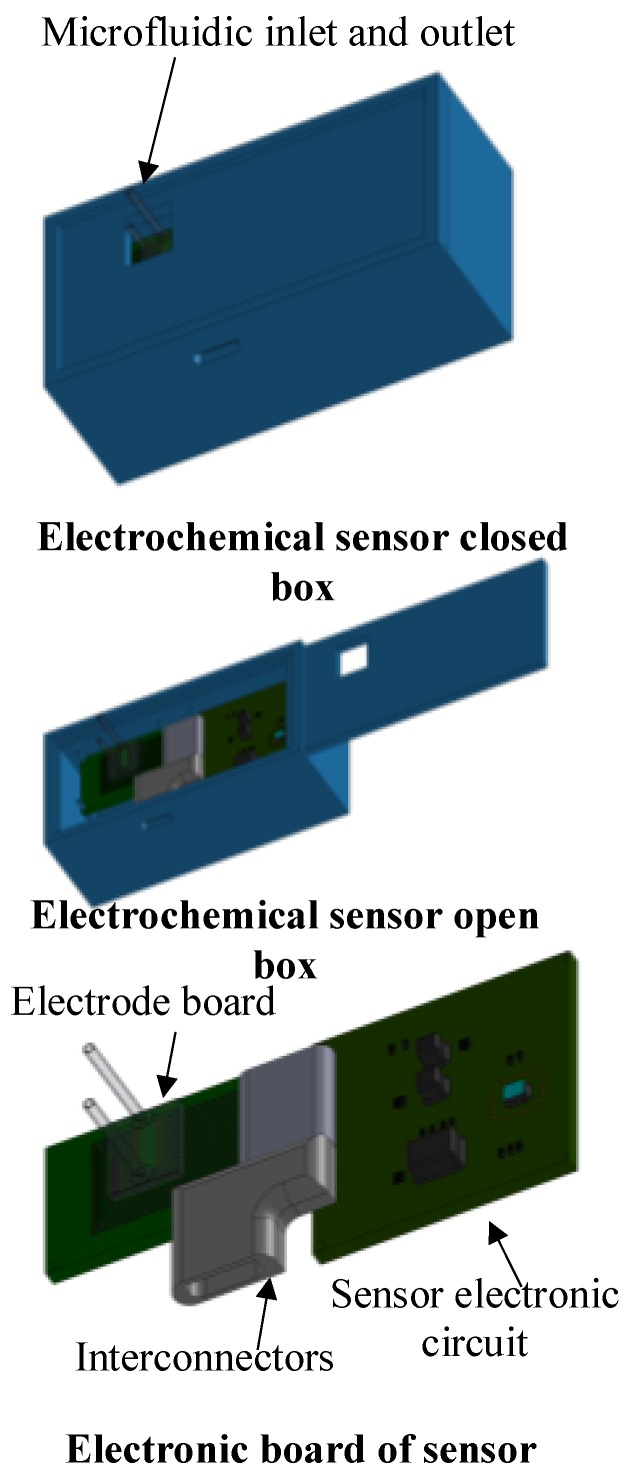
Conceptual figure of the proposed electrochemical biosensor.

Designing miniature potentiostats is highly desirable for biomedical research, but several constraints must be met; they include power consumption, chip size, sensitivity and detection limits due to low concentration of NTs [15,16,17]. In this work, we present the design and implementation of a new fully differential difference amplifier (FDDA)-based CMOS chip potentiostat that meets the aforementioned challenges for implantable applications as shown at Figure 1. The FDDA allows to eliminate several buffers and passive elements that are normally needed, thus diminishing the chip dimension and overall power consumption. In addition, it exhibits low noise, especially at low frequencies where flicker noise may be an obstacle, and covers a high dynamic range to improve the sensitivity and detection limits. This FDDA-based potentiostat was implemented in 0.18 µm CMOS and operates with a 1.8 V supply voltage.

The balance of this paper is as follows: Section 2 provides background on the single-ended differential difference amplifier (DDA) and the FDDA; Section 3 describes the proposed FDDA-based circuit architecture and Section 4 provides our validation results.

## 2. Background

A potentiostat circuit consists of a control unit, a measurement unit and two or three electrodes depending on the considered architecture [14]. It operates by keeping the voltage difference between a working electrode (WE) and a reference electrode (RE) at a value, V${}_{\mathrm{cell}}$, for which the analyte under study is able to generate a redox current. In fast-scan cyclic voltammetry, the difference voltage ramps up linearly until reaching the oxidizing voltage, and then ramps down to the initial potential, thus creating an electrochemical reaction where the NT is oxidized during the positive sweep and reduced during the negative sweep. The reaction takes place on WE while a counter electrode (CE) supplies current to the solution in order to maintain the voltage difference between WE and RE constant. The chemical reaction current is measured via WE. Typically, the measurement is made within the interval of molecule concentration where a linear relationship exists with respect to the sensed current [18].

**Figure 2:**
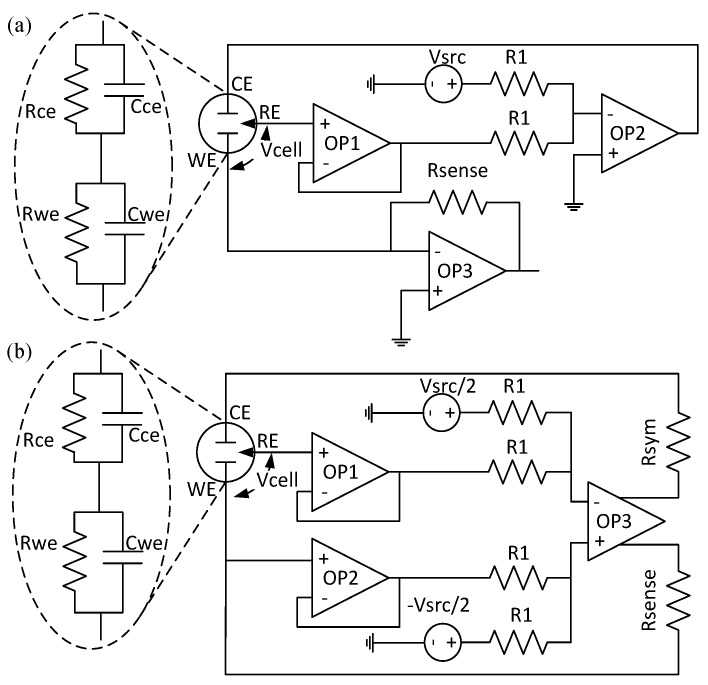
(**a**) Single-ended potentiostat diagram, showing a feedback loop to maintain V${}_{\mathrm{cell}}$ constant, and a sense resistor to convert the current generated between CE and WE electrodes to voltage; (**b**) Fully differential version for enhanced output swing.

Figure 2a shows the diagram of a single-ended (SE) potentiostat, where the virtual ground at the—input of OP2 forces V${}_{\mathrm{cell}}$ to track V${}_{\mathrm{src}}$, and OP3 senses the control current that is generated between CE and WE. Many integrated SE potentiostats have been proposed in the literature. Among recent works, Genov et al. reported a 16-channel integrated potentiostat for simultaneous monitoring of chemical neural activity at different locations in the brain [19]. Schienle et al. designed an array of potentiostats for DNA applications, while Stanacevic et al. demonstrated a 16-channel current-measuring VLSI sensor array system using a first-order single bit delta-sigma modulator with programmable oversampling ratio [20,21]. There is also Ahmadi et al. who reported a low power and small size current-mirror-based potentiostat [13].

The current advances in integrated circuit design and fabrication processes have created challenges for designing implantable SE potentiostats. Indeed, the lower operating voltages required by the smaller transistor geometry of current semiconductor technologies can violate Nernst equation, which states that the applied voltage in the solution must be higher than the oxidoreduction voltage of the eletroactive substance for a reaction to take place [22]. One approach to maintain sensitivity while applying a low supply voltage is to use a fully differential (FD) architecture to widen the voltage swing of the potentiostat (in comparison to an SE architecture). An FD potentiostat also solves the performance degradation of SE potentiostats due to common-mode interference [23]. There have been several FD architectures proposed in the literature in the past years. For instance, Wang et al. [24] implemented an FD potentiostat that operates in differential signal mode for the output voltage. It shows low power consumption, low-to-medium operation frequency and medium-to-high resolution that are suitable for a portable potentiostat. Martin et al. [14] described a FD potentiostat that doubles the dynamic range of the system, but more importantly, maximizes the number of detectable analytes for a given supply voltage. Finally, Nazari et al. [23] designed a potentiostat that is able to detect analytes directly via on-chip recording electrodes while benefiting from differential recording electrodes to suppress common mode noise [23].

Figure 2 shows the block diagrams of the three-electrode SE and FD potentiostats [14]. In this figure, C${}_{\mathrm{CE}}$ and C${}_{\mathrm{WE}}$ stand for the double-layer capacitance of CE and WE, while R${}_{\mathrm{CE}}$, R${}_{\mathrm{WE}}$ represent the charge transfer resistance of the relevant electrodes. The current to be measured is monitored through a sensing resistor, R${}_{\mathrm{sense}}$, with R${}_{\mathrm{sym}}$ added for symmetric operation in the FD case [14]. We have for both SE and FD circuits:

(1) $$V_{src}=V_{WE}-V_{RE}=V_{cell}$$

(2) $$V_{out}=I_{sense}R_{sense}$$

Therefore, both potentiostats ensure that V${}_{\mathrm{cell}}$=V${}_{\mathrm{src}}$, but the voltage swing of the FD potentiostat is nearly twice that of the SE case, as shown next [14]:

(3) $$S_{SE}=V_{dd}\frac{R_{WE}}{R_{WE}+R_{CE}}$$

(4) $$S_{FD}=\left|V_{dd}-V_{ss}\right|\frac{R_{WE}}{R_{WE}+R_{sense}+R_{sym}+R_{CE}}$$

In the previous equations, $S_{SE}$ is the output swing voltage of single-ended amplifier and $S_{FD}$ is the output swing voltage of fully-differential amplifier.

Equations (3) and (4) reveal that for both the SE and FD potentiostat, the maximum voltage swing is bounded by the supply voltage term, but it is doubled in the differential architecture [25,26,27]. In addition, the reduced common mode performance of the FD architecture considerably improve the sensitivity and detection limits of the potentiostat. However, power consumption can be a problem due to the increased circuit complexity. One approach to address this issue is to reduce the number of amplifiers and resistors in the front end by using a differential difference amplifier (DDA), which already contains two differential pairs of inputs (four inputs instead of two in a conventional FD amplifier). Moreover, an FD version of DDA, called FDDA, can be used for improved output voltage swing. The FDDA improves the input differential range, supply noise rejection, dynamic range, harmonic distortion and reduces the effect of coupling between various blocks [28,29]. Additionally, a rail-to-rail input/output swing maximizes the output dynamic range for the potentiostat, thus offering a wider range of voltages for NT detection [14]. The balanced outputs of the FDDA are given in Equation (5), in which A${}_{O}$ is the open-loop gain of the FDDA, V${}_{\mathrm{pp}}$ and V${}_{\mathrm{pn}}$ are the non-inverting pairs, and V${}_{\mathrm{np}}$ and V${}_{\mathrm{nn}}$ are the inverting pairs:

(5) $$V_{op}=\;-V_{on}=\;A_{O}\left[\left(V_{pp}-V_{pn}\right)\;-\;\left(V_{np}-V_{nn}\right)\right]$$

When a negative feedback loop is created with the FDDA, the two differential input pairs become equal as shown in Equation (6):

(6) $$V_{pp}-V_{pn}=\;V_{np}-\;V_{nn};\;A_{O}\to \;\infty$$

## 3. Proposed Circuit Architecture

**Figure 3:**
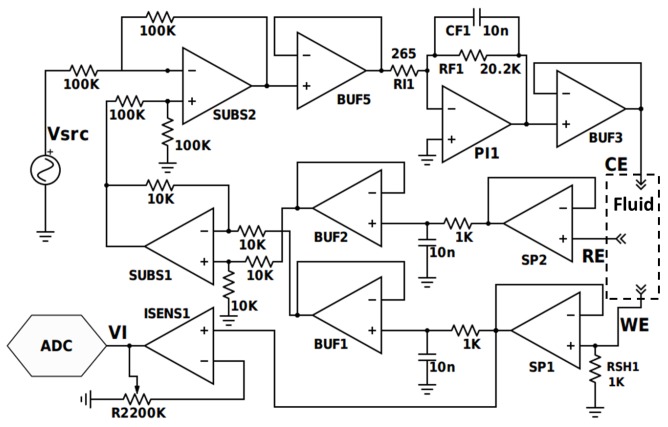
Initial version of the potentiostat design.

A first version of our potentiostat, shown in Figure 3, was designed with discrete components using 10 operational amplifiers, 14 off-chip resistors, and 3 large capacitors (10 nF). An optional ADC was added for data acquisition, and the system was implemented on a 4.5 cm × 3.3 cm PCB. This version is fully functional, enabling the detection of dopamine with concentrations down to 10 µM. This design is independent from the fluid’s impedance since it measures the voltage across the load using the buffered inputs SP1 and SP2 and compensate for any error using the buffered output BUF3 using error integration. Amplifiers BUF1, BUF2, SUBS1 and SUBS2 are used to calculate the error: $V_{err}=V_{src}-\left(V_{RE}-V_{WE}\right)$ between the desired voltage $V_{src}$ and the actual voltage applied on the fluid. BUF5, PI1 and BUF3 are used to compensate the error and maintain the desired voltage across the fluid. However, a problem arises with this circuit when measurements are desired for implantable bio-application: the number of electrical components needed to implement this design on-chip is too high. Since the number of amplifiers used in this design can hardly be reduced to keep the exact functionality, we investigated another approach based on FDDA in order to miniaturize the circuit toward an implantable device for bio-application. Although fluid impedance is now part of this design feedback loop calculations, the low number of components allows an optimized miniaturization.

**Figure 4:**
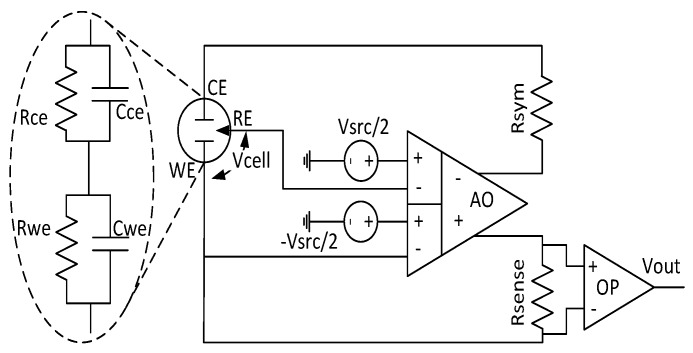
Bloc diagram of the proposed FDDA-based FD potentiostat.

Figure 4 provides the schematic of the proposed FDDA-based potentiostat. Based on Equation (6), the negative feedback used for this circuit leads to the following equations:

(7) $$V_{src/2}-V_{WE}=\;-V_{src/2}\;-V_{RE}$$

(8) $$V_{src}=\;V_{WE}\;-V_{RE}=\;V_{cell}$$

As already mentioned, R${}_{\mathrm{sense}}$ converts the redox current to current and R${}_{\mathrm{sym}}$ is added to balance the FDDA output. Obviously, choosing high R${}_{\mathrm{sense}}$ values will allow to sense low redox currents and, consequently, low analyte concentrations.

However, high R${}_{\mathrm{sense}}$ values also lead to high-voltage drops across this resistor, with a negative impact on the system accuracy and linearity. Moreover, for best sensitivity, R${}_{\mathrm{sense}}$ cannot be increased without regard for the injected noise in the sensed current, which must be as low as possible.

The sensing current should only flow from CE to WE, as it will affect the RE voltage otherwise. The common method to prevent this is to buffer the RE electrode as shown in Figure 2b. This is achieved by default in the proposed potentiostat, where the RE terminal connects to the gate of a MOSFET in the FDDA, hence saving an operational amplifier and lowering the overall power consumption as a result.

**Figure 5:**
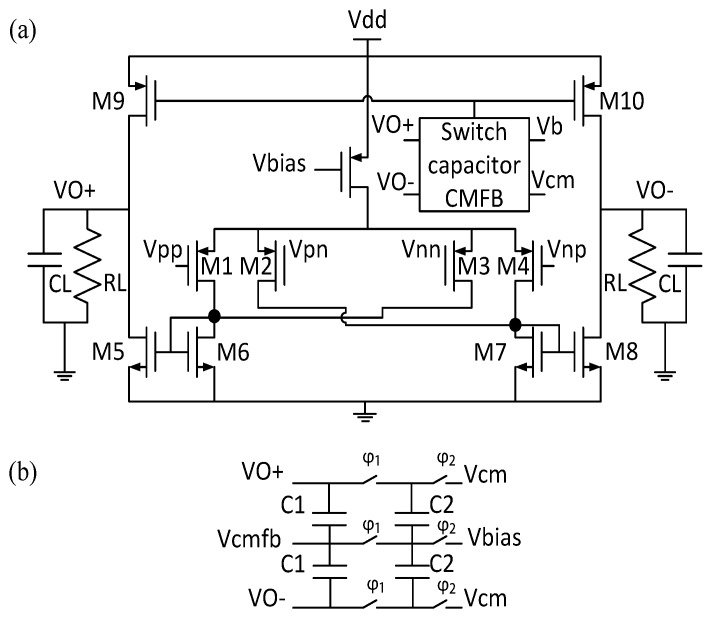
(**a**) Component-level schematic of the FDDA bloc A${}_{0}$ of Figure 4, including the switch-Capacitor CMFB shown in (**b**).

The circuit schematic of the proposed FDDA is shown in Figure 5. It is adapted from [28] and relies on a four-input, two-stage, current-mirror-based architecture. In this design, input transistors (M${}_{1}$–M${}_{4}$) are selected with the same dimensions of width (W) and length (L); transistors M${}_{5}$–M${}_{8}$ and M${}_{9}$–M${}_{10}$ also have the same in size. Additionally, a common-mode feedback (CMFB) circuit to tune the common-mode output voltage is implemented with switched capacitors. The CMFB circuit is also used to adjust the dc value of the differential outputs via V${}_{\mathrm{bias}}$.

One important parameter to consider in the FDDA is its input-referred noise. Its average mean-squared value is given by [30]:

(9) $$\bar{V_{ni,thermal}^{2}}=\left[\frac{16kT}{3}\left(\frac{2}{{g_{m}}_{1}}+\frac{2{g_{m}}_{5}}{{{g_{m}}_{1}}^{2}}+\frac{{g_{m}}_{9}}{{{g_{m}}_{1}}^{2}}\right)\right]\Delta f$$

From Equation (9), minimum thermal input-referred noise can be achieved by maximizing g${}_{m_{1-4}}$ and minimizing g${}_{m_{5-8}}$ and g${}_{m_{9-10}}$. In order to augment the small-signal transconductance of M${}_{1-4}$ (g${}_{m_{1-4}}$) and decrease g${}_{m_{5-10}}$, (W/L)${}_{1-4}$ should be as big as possible while (W/L)${}_{5-10}$ should be as small as possible. By taking into account the chosen transistor dimensions and applying a low bias current, M${}_{1}$–M${}_{4}$ will operate in the weak inversion (subthreshold) region, and M${}_{5}$–M${}_{8}$ and M${}_{9}$–M${}_{10}$ will operate in the moderate inversion region. The bias current of the input stage can be set by off-chip resistors connected to the V${}_{\mathrm{Bias}}$ input (We estimated a risk of up to 20% drift in resistance value due to fabrication process if these resistors were embedded on chip. Therefore, we preferred to use off-chip resistor in the first version of the prototype for better monitoring of chip performances).

Another important FDDA noise parameter to consider is the flicker noise (1/f noise). Since most chemical reactions occur at low frequencies, lowering this noise is critical in a potentiostat design. For the circuit of Figure 5, the average mean-squared value of the flicker noise is given by [30]:

(10) $$\begin{array}{cc} \bar{V_{ni,flic\ker}^{2}} & =\frac{2}{C_{ox}f}[\frac{4K_{p}}{W_{1}L_{1}}+\frac{4K_{n}}{W_{5}L_{5}}{\left(\frac{g{{}_{m}}_{5}}{g{{}_{m}}_{1}}\right)}^{2}\\ + & \frac{2K_{p}}{W_{9}L_{9}}{\left(\frac{g{{}_{m}}_{9}}{g{{}_{m}}_{1}}\right)}^{2}]\Delta f \end{array}$$

where K${}_{n}$ and K${}_{p}$ are the flicker noise constants for nMOS and pMOS transistors, respectively, and C${}_{\mathrm{ox}}$ is the gate oxide capacitance per unit area. Equation (10) shows that the flicker noise contribution of M${}_{5}$–M${}_{10}$ is negligible when g${}_{m_{1}}$≫g${}_{m_{i}}$, leaving transistors M${}_{1}$–M${}_{4}$ as the main contributors to flicker noise in the circuit; pMOS transistors are used to take advantage of their lower flicker noise in comparison to nMOS transistors [29,30,31]. To reduce the flicker noise, M${}_{1}$–M${}_{4}$ should be as wide as possible and M${}_{5}$–M${}_{10}$ should be as narrow as possible, but circuit stability must be considered when doing so. For a stable system (phase margin ≥ 45${}^{\circ }$), the two poles frequencies at g${}_{m_{6}}$/C${}_{6}$ and g${}_{m_{7}}$/C${}_{7}$ should be higher than the dominant pole at g${}_{m_{1}}$/C${}_{L}$, where C${}_{5}$–C${}_{9}$ are the gate capacitances of transistors M${}_{5}$–M${}_{9}$ and C${}_{L}$ is the load capacitor, whose value depends on the chemical cell and electrodes used. So, in order to lower noise in the system, g${}_{m_{5}}$ and g${}_{m_{9}}$ cannot be reduced arbitrarily (by using large a C${}_{L}$ and low g${}_{m_{1}}$ and g${}_{m_{9}}$). Indeed, (g${}_{m_{1}}$/C${}_{L}$) ≪ (g${}_{m_{9}}$/C${}_{9}$, g${}_{m_{5}}$/C${}_{5}$) when C${}_{L}$ ≫ C${}_{9}$, C${}_{5}$ [30].

Finally, we must also consider the FDDA open-loop gain, which can be increased by current mirror amplification between the first and second stages. Here, unit-gain current mirror amplification is used to meet the constrains of Equations (9) and (10). The gain of the proposed FDDA is given by [30]:

(11) $$\begin{array}{cc} A_{V} & =\frac{1}{4}g_{m_{1}}\left(\frac{g_{m_{5}}}{g_{m_{6}}}\right)(R_{L}\left|\right|R_{ds9}\left|\right|R_{ds5})\\ + & \frac{1}{4}g_{m_{3}}\left(\frac{g_{m_{5}}}{g_{m_{6}}}\right)(R_{L}\left|\right|R_{ds9}\left|\right|R_{ds5})\\ + & \frac{1}{4}g_{m_{2}}\left(\frac{g_{m_{8}}}{g_{m_{7}}}\right)(R_{L}\left|\right|R_{ds10}\left|\right|R_{ds8})\\ + & \frac{1}{4}g_{m_{4}}\left(\frac{g_{m_{8}}}{g_{m_{7}}}\right)(R_{L}\left|\right|R_{ds10}\left|\right|R_{ds8}) \end{array}$$

As the dimensions of M${}_{1}$–M${}_{4}$, M${}_{5}$–M${}_{8}$ and M${}_{9}$–M${}_{10}$ are identical and R${}_{L}$ is much smaller than R${}_{\mathrm{ds}}$ (drain-source resistor) for M${}_{9}$–M${}_{10}$ and M${}_{5}$–M${}_{8}$, Equation (11) can be rewritten as Equation (12). As a result, the design benefits from the high value of g${}_{m_{1}}$ to generate the appropriate negative feedback gain.

(12) $$A_{V}=4\times \frac{1}{4}g_{m_{1}}\left(\frac{g_{m_{5}}}{g_{m_{6}}}\right)\left(R_{L}\right)=g_{m_{1}}R_{{}_{L}}$$

In order to achieve a low-noise circuit, the input stage transistors were designed with a width/length ratios in the range of 200/1. However, this large ratio normally leads to more power consumption. Using a low bias current and operating the input stage transistors in the weak inversion mitigates this effect.

The reported input-referred noise (µV${}_{\mathrm{rms}}$) includes the CMFB noise. For low concentrations, the CMFB noise is an obstacle to sensitivity due to its clock interference with the sensed current. Fortunately, the clock frequency was 1 MHz, while the input signal bandwidth was 0–100 Hz, thus allowing to eliminate this noise a simple RC low-pass filter with cut-off frequency close to 100 Hz.

## 4. Experimental Verification and Validation

Two types of experiments were conducted, one to verify the system operation and the other to validate it in vitro. Next is a description of the two.

### 4.1. System Verification

**Figure 6:**
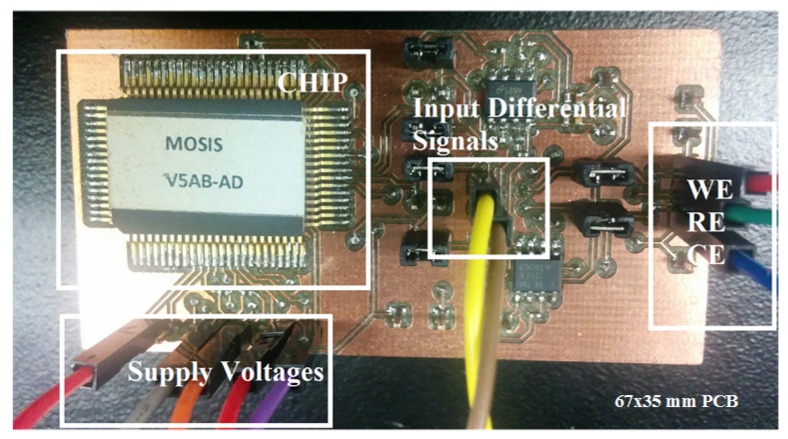
Assembled potentiostat with CQFP48 ceramic package on PCB including electrode interface, supply voltage and input signals.

**Figure 7:**
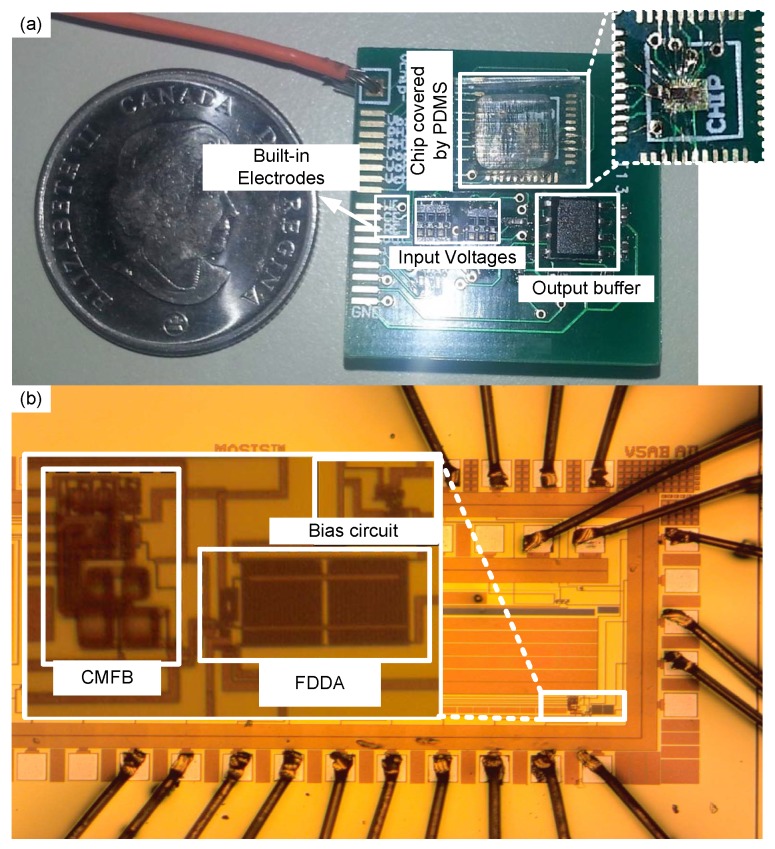
(**a**) Potentiostat system assembled on 30 mm × 30 mmm PCB connected to electrodes, supply voltages and input signals, (**b**) microphotograph of the embedded chip including biasing circuit of the the CMOS chip with FDDA and CMFB layout.

A first prototype of 67 mm × 35 mm dimensions was designed to test the functionality of the system with non-embedded electrodes. A second system was designed on a 20 mm × 20 mm PCB with embedded electrodes and a microfluidic chamber fo size 8 mm × 8mm. The fabricated chip area by itself was about 1 mm × 0.5 mm. A first prototype of the on-chip potentiostat described in the previous section was designed in 180 nm CMOS technology, with the resulting chip encapsulated in a CQFP48 ceramic package. In addition, the transistors placed close to the chip edges, which are more sensitive to fabrication process mismatches, were increased in size a bit. All the resistors were designed for off-chip placement to improve control of the bias current and minimize the mismatch impacts. The chip was soldered on a PCB (67 mm × 35 mm) illustrated in Figure 6 to make possible the application of a 0 to 1.8 V triangular waveform to the V${}_{\mathrm{pp}}$ and V${}_{\mathrm{np}}$ inputs, and two non-overlapping 1 MHz differential clocks to the switched-capacitor CMFB circuit. Using 1 MHz clock frequency was sufficient for the proper operation in comparison to the 100 Hz frequency of the differential input signals [30] (the cyclic voltametry analysis in our experiments used 100 Hz frequency). R${}_{\mathrm{sense}}$ was set to 1 MΩ to allow the detection of redox currents with high linearity. Figure 7b shows the microphotograph of the fabricated chip using the 180 nm CMOS process of TSMC, with the bias, FDDA and CFMB parts identified.

Following the first prototype, second compact PCB prototype (25 mm × 25 mm) was designed where the potentiostat chip was wire-bonded directly on the PCB as a loose dye, and where An 8 mm × 8 mm polydimethylsiloxane (PDMS) chamber was designed to cover and protect the CMOS chip from liquid leakage. Figure 7b shows a picture of the second prototype.

As already mentioned, the only physical contact of the potentiostat with a solution is through electrodes WE, CE and RE, and the generated redox current passes through WE to induce a voltage difference across R${}_{\mathrm{sense}}$. An external low-bias-current and low-noise instrumentation amplifier (INA121 FET-Input, Texas Instrument, USA) buffers this voltage while providing additional gain if needed. (shown in Figure 6 and Figure 7a).

Since the feedback loop of the potentiostat includes the chemical solution as well as electrodes WE, CE and RE, the area and ruggedness of these electrodes, and the analyte concentration, considerably affect the system accuracy and sensitivity [32]. In this work, the electrodes surfaces were covered with gold by electro-deposition, with an estimated plating thickness of 1 µm [16,33]. A 99.99% solution of chloroauric acid (HAuCl4) was used.

**Figure 8:**
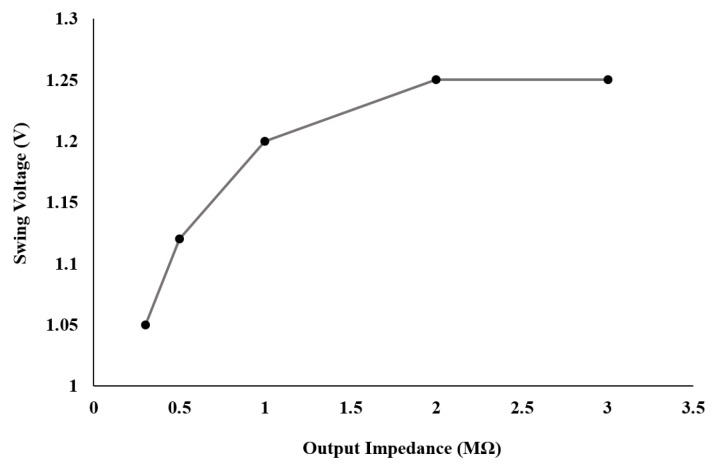
Output swing voltage over output impedance with a fully resistive load.

Before reporting the system performance with different solutions, we show in Figure 8 the impact of the output impedance on the output voltage swing. A stable system response can be observed down to 500 kΩ. (we define as stable a linear response for voltage swing, with no limiting effects when increasing the input voltage in the closed loop). Below that value, we are below the 90% of 1.25 V, the threshold for a stable potentiostat behavior with regards to the swing voltage.

**Figure 9:**
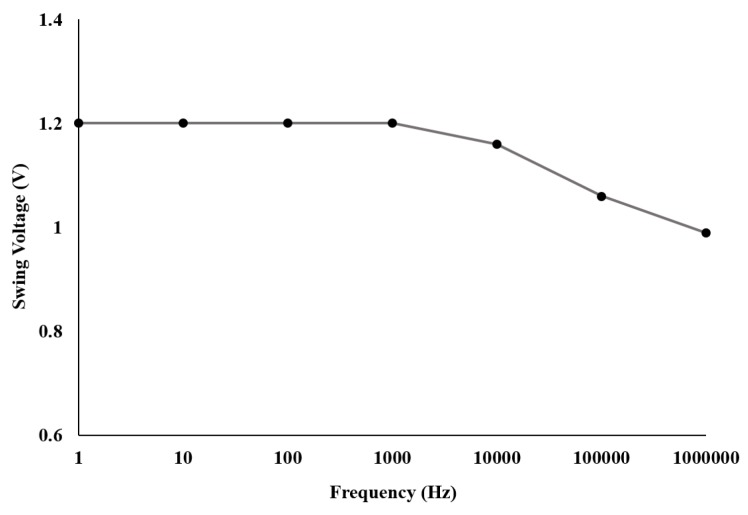
Output swing voltage for different frequencies, where a stable behaviour has been observed at frequencies below 1 kHz.

The frequency response of the closed-loop output voltage swing of the potentiostat was also analyzed. Figure 9 presents the obtained results, showing a inverse relationship between the output voltage swing and frequency, except for an initial plateau that ends at 1 kHz. In our intended applications, the fabricated potentiostat operates mostly below 100 Hz, hence at the maximum voltage swing.

**Figure 10:**
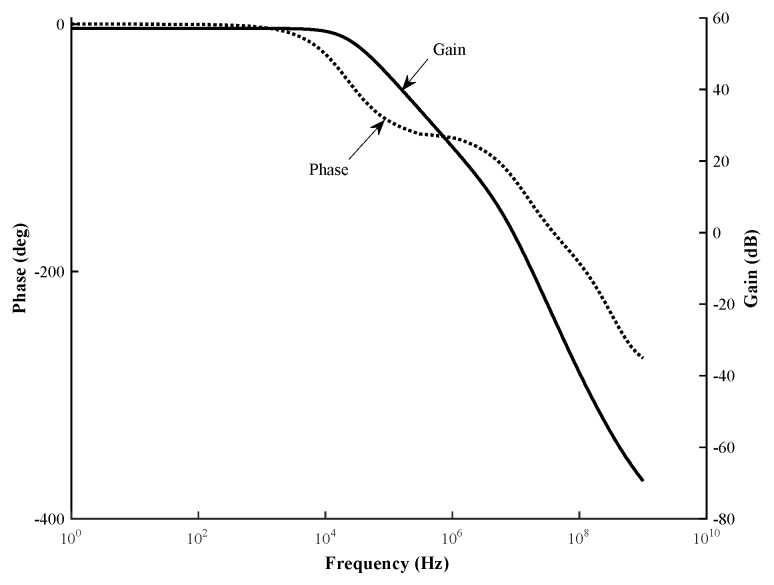
Simulation results of gain and phase figure of designed FDDA based architecture of the potentiostat.

The other performances of the fabricated potentiostat were a dc gain of 57 dB and a unity-gain bandwidth of 9 MHz, with a phase margin of 55${}^{\circ }$ as shown at Figure 10. The slew rate was 21 V/µs, and the input differential range and input offset voltage of the FDDA were 1 mV and 14 µV, respectively. The measured power consumption of the chip was 53.85 µW using a 1.8 V supply voltage, with the FDDA and CMFB accounting for 33.69 µW and the voltage bias circuit for 20.16 µW. Finally, the common-mode rejection ratio (CMRR) was 84 dB, and the cut-off frequency 10 kHz.

**Figure 11:**
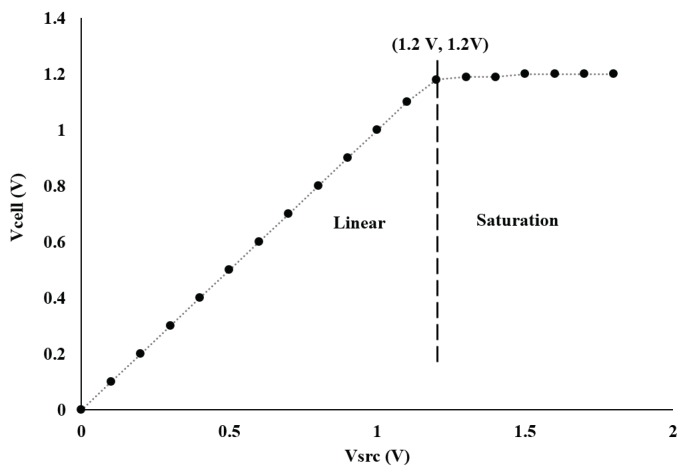
Performance of the first prototype in term of linearity and saturation where linear behavior is observed below 1.2 V.

The potentiostat circuit was also tested in closed loop to analyze its output voltage swing. As shown in Figure 11, the chip’s current sourcing capability saturates when the applied input voltage is more than 1.2 V. Therefore, the maximum differential output voltage range is 2.4 V when the chip is supplied with 1.8 V.

**Figure 12:**
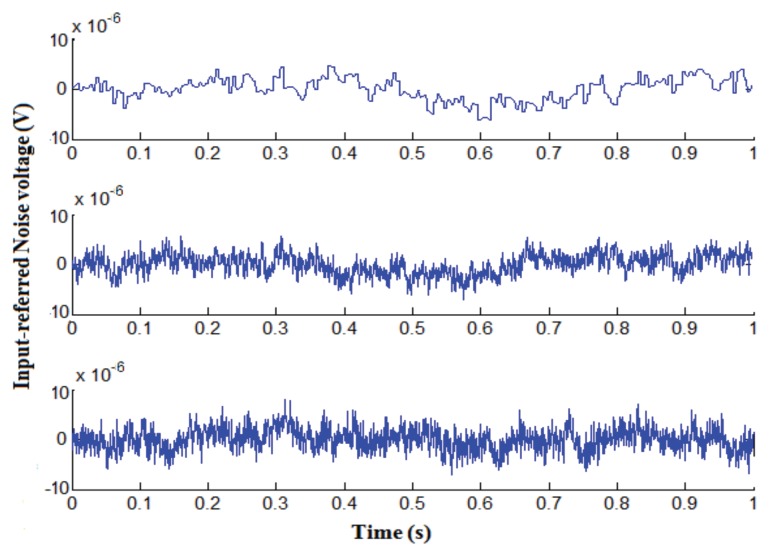
Input-referred noise voltage of approximately 6.9 $\mu V_{\mathrm{rms}}$ for frequencies of (**Top**) f = 100 Hz (**Middle**) f = 1 KHz (**Bottom**) f = 10 KHz.

To evaluate the noise performance of the potentiostat, the inputs were grounded and transient analysis was performed. The obtained output RMS noise amplitude was then divided by the amplifier gain (707 V/V), yielding 6.9 µV${}_{\mathrm{rms}}$ for the input-referred noise. Figure 12 shows three sample noise records at 100 Hz, 1 kHz and 10 kHz operating frequencies. As can be seen, obtained data look similar, which indicates that flicker noise is not dominant in this architecture, and the system can be operated at low frequency with no adverse effect due to this noise. Indeed the difference between three figures does not exceed 1 µV${}_{\mathrm{rms}}$.

### 4.2. In Vitro Validation

We used ferricyanide (Sigma-Aldrich, St. Louis, MO, USA) for system calibration. Ferricyanide was chosen for its stable electrochemical properties. After calibration, we conducted additional experiments to test the proposed potentiostat on an actual NT. Dopamine (Sigma-Aldrich, St. Louis, MO, USA) was used for the purpose, as it is highly electroactive.

**Figure 13:**
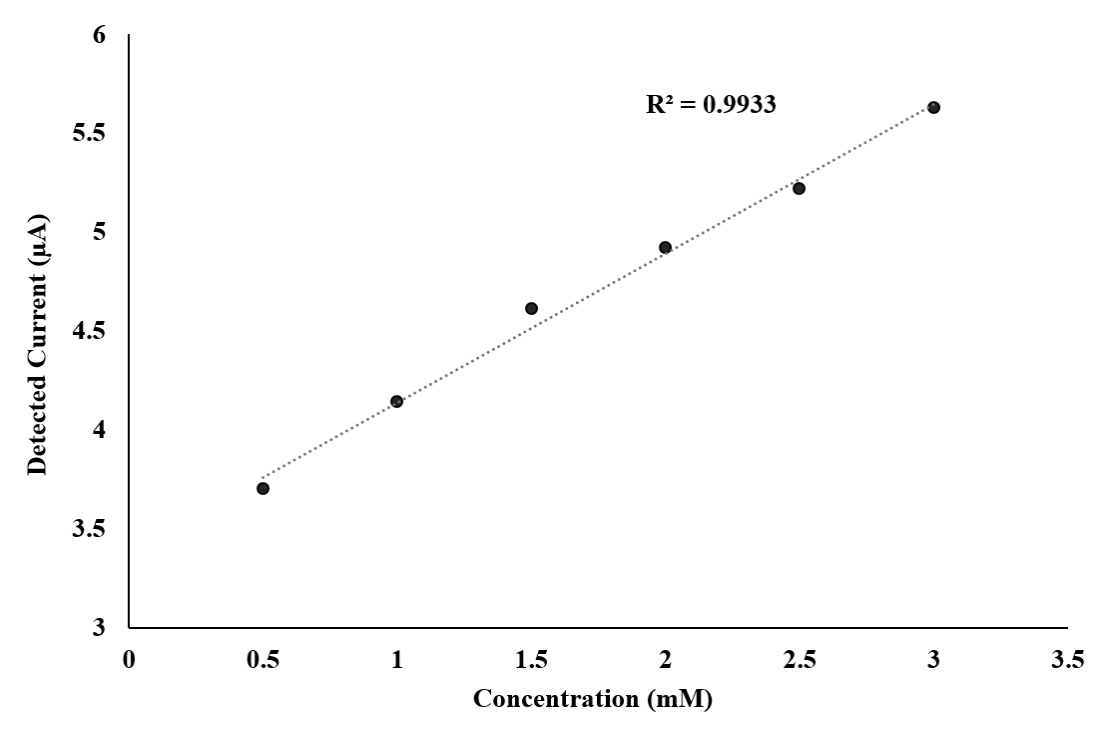
Sensitivity and linearity of implemented chip analyzing reduction peaks of ferricyanide over different concentrations with R${}_{\mathrm{sense}}$ = 1 MΩ.

The electrochemical analysis of ferricyanide was carried out with solutions from 500 µM to 3 mM, to determine the sensitivity and linearity of the system. The highest detected concentration without saturating the system was 3 mM. Figure 13 provides the system sensitivity for different concentrations of ferricyanide. As can be seen, the sensitivity of the potentiostat was in the micro-ampere range, and the 0.9933 regression factor attests of the high system linearity.

**Figure 14:**
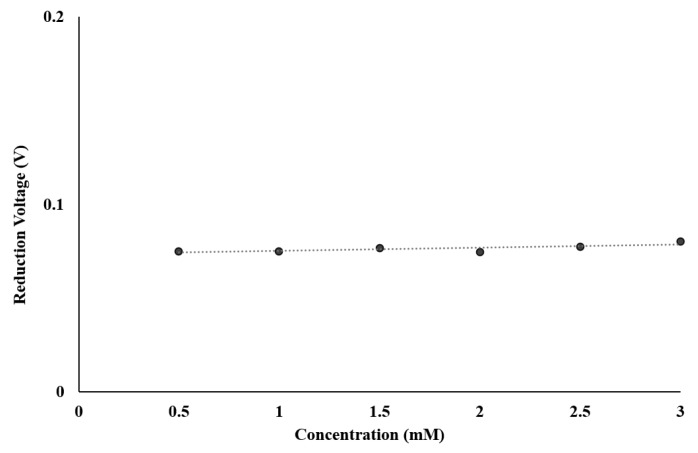
Reliability of design chip in term of reduction peaks of Ferricyanide over different concentrations with R${}_{\mathrm{sense}}$ = 1 MΩ.

The input voltage at which the reduction occurs for different concentrations was also measured to test the system’s operational consistency. Figure 14 shows that in all tests, the redox peaks were occurring at almost identical voltage of 75 mV, thus proving a high operational consistency.

**Figure 15:**
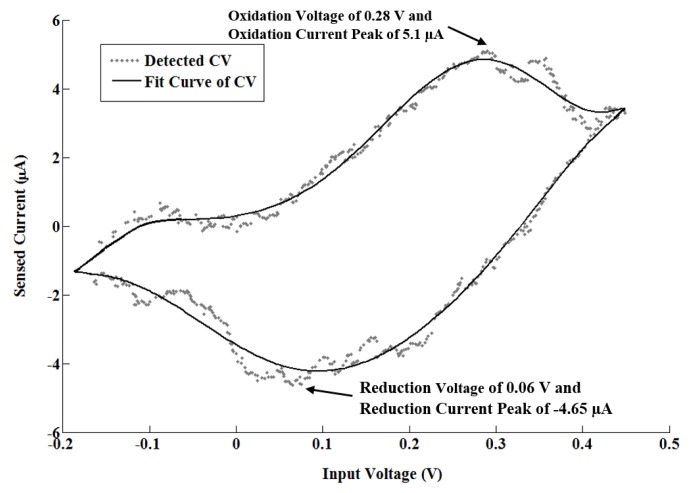
Example of cyclic voltammetry analysis obtained with 1 mM Dopamine with R${}_{\mathrm{sense}}$ = 1 MΩ.

**Figure 16:**
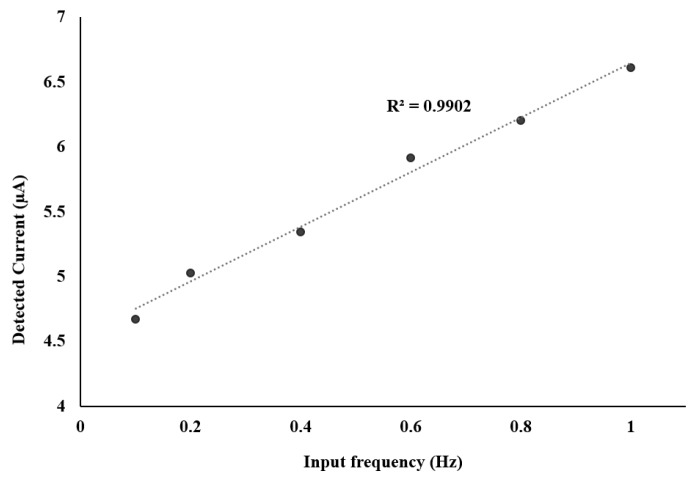
Effect of frequency on sensitivity of implemented designed potentiostat with R${}_{\mathrm{sense}}$ = 1 MΩ.

After verification with ferricyanide, different concentrations of a dopamine solution, from 1 mM to 10 mM, were used in order to test the potentiostat response to a neurotransmitter. Figure 15 reports an example of obtained CV with dopamine at a concentration of 1 mM and R${}_{\mathrm{sense}}$ = 1 MΩ. Figure 15 shows the CV response with a scan rate of 100 mV/s. The reduction and oxidation peaks correspond to currents of −5 µA and 5 µA, respectively, with an input voltage from −400 mV to 300 mV. Figure 16 shows the corresponding detected current as a function of scan rate (labeled input frequency). As expected, the minimum detected current increased with frequency. The figure also shows a regression factor of 0.9902 and a sensitivity at the order of micro-amperes.

We set the system to work continuously without any standby mode, and static power consumption was not optimized to the minimum. The linearity of the system was also very high (>0.99). On the other hand, other version of the prototype is being developed to improve the sensitivity in term of electronic and electrode design. In this regard, more effort should be devoted to optimizing the electrode arrangement and surface areas.

As shown in Table 1, the accuracy of our potentiostat is 0.993 and it uses only one FDDA amplifier, for a current sensitivity of 60 µA at the concentration of 1 µM with screen printed electrodes. In addition, our chip has relatively lower power consumption, since the ones reported in the other references may be related to complete system designs and multi-channel potentiostats. It should be noted also that this is the first iteration of the design, in which our main focus is the number of amplifier needed and accuracy in terms of linearity. The obtained current noise performance is acceptable (7 pA) for this design as we are not targeting concentration lower than 1 µM, which is equivalent to a sensing current of 60 µA. This system was designed as proof of concept for in-vitro experiments with artificial cerebrospinal fluid. It was not optimized for real implants.

The smallest concentration of dopamine that we were able to detect with was 1 mM. When the concentration is more than 3 mM the output was saturated and when it was below 500 µM there was interference with clock of CMFB. This is due to several factors, of which the potentiostat electrodes. We used electroless gold plating for them, which did not allow us a strong control over the quality of deposition. Therefore, the electrode surface area could not be accurately determined, thus preventing us from testing smaller dopamine concentrations. Other electrodes based on fiber are being fabricated and they should lead to higher sensitivity. On the other hand, obtained results show a strong reliability of our system, since the gold-plated electrodes were tested for two months with repeatable results. Afterward, the gold layer started disappearing.

**Figure 17:**
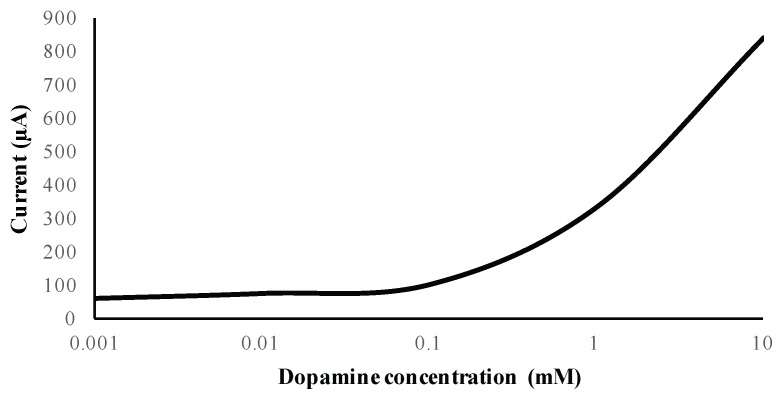
Linearity of designed biosensor with dopamine (1 µM to 10 mM).

**Figure 18:**
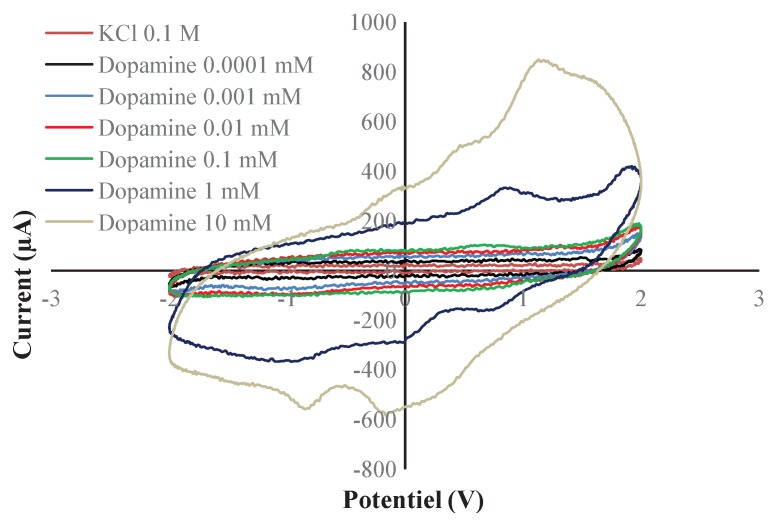
Cyclic voltamogrammes for dopamine with 0.1 M KCl solution as solvant. Concentration of dopamine varies between 0.1 µM and 10 mM.

Different electrode architectures were tested to evaluate the system sensitivity to dopamine. We used dopamine with a KCl solution as solvent. We observed two behaviors as shown in Figure 17 and Figure 18. Indeed as can be seen in Figure 17, when the dopamine was between 1 µM and 0.1 mM, the detected current varied between 60 µA and 100 µA. On the other hand, when the concentration was between 0.1 mM and 10 mM, the detected current was in the range of 100 µA to 840 µA. Thus, we observed two linear behaviors: one with 0.074 µA/mM at high concentration and 404.04 µA/mM at low concentration. We used carbon screen printed electrodes (RRPE1002C from PINE research, Durham, NC, USA). The stimulation voltage out of the chip was amplified with a commercial amplifier to cover the entire range from –2 V to 2 V, order to make sure that the oxidation peaks are accounted for. The experimental results clearly show that they did not exceed 1.13 V, which is within the operating voltage range of the designed chip. In terms of detected concentration range, the obtained results are similar to those reported in [34]. In terms of electrical performance, the power consumption of our system is 53 µW, versus 25 mW for 576 electrodes in [35,36]. A coarse approximation of the latter mentioned work leads to a power consumption of (25/576) mW/channel which is equivalent to 43.4 µW. Therefore, we are in the same range of power consumption. However, this is a coarse approximation as some circuits are shared between different channels in the case of [35,36].

DDA and FDDA circuits have four inputs with no need for passive elements, which make them attractive for low power and low noise applications. FDDA is the fully differential architecture of the DDA, for improved input differential range, supply noise rejection, harmonic distortion and reduced coupling effects between the various circuit blocks. Also, increasing the output swing improves the output dynamic range and can cover a wider range of detected neurotransmitters. On the other hand, a potential drawback to using the FDDA is the CMFB circuit to control the common-mode output voltage, which introduces clock interferences. Overall, using FDDA architecture reduces power consumption and noise level while improving the dynamic range and output voltage swing.

Furthermore, the overall dimensions of the system were not optimized in the first prototype, where we used wire-bonding instead of more area-efficient packaging techniques such as flip-chip, and we placed the off-chip resistors further away from the chip than needed, for probing and debugging purposes. Another CMOS TSMC 180 nm chip was submitted for fabrication that includes all data acquisition amplifiers in order to reduce the biosensor area as much as possible. If we compare our die area to other published work, our area of 1 mm × 0.5 mm is smaller than [3,19] which reports 2.25 mm × 2.25 mm for 16 channels and 3.8 mm × 3.1 mm for 93 channels. However, it should be noted that in the previous example we are comparing die sizes and not one-channel circuit area.

The concentration of dopamine varies across the brain, hence motivating the need for wide dynamic range potentiostat. The system described in this work was designed primarily for in vitro experiments with artificial solutions and brain slices. A such, it was not optimized for real brain application. Currently, a second iteration is being investigated to sense concentrations down to nM and a prototype has been sent for fabrication to check its validity.

## 5. Conclusions

This paper presented a new potentiostat topology based on a FDDA circuit. The chip was fabricated with the 180 nm TSMC CMOS process. This FDDA-based potentiostat used four-input and FD output to decrease the number of amplifiers and passive elements in the circuit and to decrease the generated noise through the circuit. Moreover, The chip is designed with a wide input stage to benefit from the diminished input-referred noise of 6.9 µV${}_{\mathrm{rms}}$. The whole decreased noise in both the circuit and system levels improved the sensitivity and accuracy of the potentiostat. The FD output is an original approach to detect the analytes. The main advantage of this architecture is the doubled swing voltage over V${}_{\mathrm{cell}}$. Additionally, due to current-mirror architecture of the designed FDDA, the total power consumption of the potentiostat was 53.9 µW. The system was verified and tested with ferricyanide and dopamine. Further improvements in terms of power consumption are being achieved in addition to tests with other NTs.

## Figures and Tables

**Table 1:** Comparison Table.

	Nazari [3]	Martin [14]	Ahmadi [17]	Mamun [33]	Our work
Technology	0.35 µm	0.18 µm	0.18 µm	0.18 µm	0.18 µm
Supply voltage	3.3 V	1.8 V	1.8 V	1.8 V	1.8 V
Sensitivity	24 pA	5 nA	N.A	100 nA	5 µA
Power consumption	188 µW	15.84 mW	171 µW	72.36 µW	**53 µW**
Accuracy	N.A	0.998	N.A	N.A	**0.993**
Input-referred noise	N.A	N.A	38 µV	N.A	**6.9 µV${}_{\mathrm{rms}}$**
Number of amplifiers	N.A	3	1	N.A	1
Architecture	FD	FD	SE	DD	**FDD**

## References

[1] Mulliken G., Naware M., Bandyopadhyay A., Cauwenberghs G., Thakor N. Distributed neurochemical sensing: In vitro experiments. Proceedings of the IEEE 2003 International Symposium on Circuits and Systems (ISCAS’03).

[2] Strong T.D., Martin S.M., Franklin R.F., Brown R.B. Integrated electrochemical neurosensors. Proceedings of the 2006 IEEE International Symposium on Circuits and Systems.

[3] Nazari M.H., Mazhab-Jafari H., Leng L., Guenther A., Genov R. (2013). CMOS neurotransmitter microarray: 96-Channel integrated potentiostat with on-die microsensors. IEEE Trans. Biomed. Circuits Syst..

[4] Murari K., Thakor N., Stanacevic M., Cauwenberghs G. Wide-range, picoampere-sensitivity multichannel VLSI potentiostat for neurotransmitter sensing. Proceedings of the 26th Annual International Conference of the IEEE Engineering in Medicine and Biology Society (IEMBS’04).

[5] Linert W., Jameson G. (2000). Redox reactions of neurotransmitters possibly involved in the progression of Parkinson’s Disease. J. Inorg. Biochem..

[6] Perry M., Li Q., Kennedy R.T. (2009). Review of recent advances in analytical techniques for the determination of neurotransmitters. Anal. Chim. Acta.

[7] Mollazadeh M., Murari K., Cauwenberghs G., Thakor N. (2009). Wireless micropower instrumentation for multimodal acquisition of electrical and chemical neural activity. IEEE Trans. Biomed. Circuits Syst..

[8] Gore A., Chakrabartty S., Pal S., Alocilja E.C. (2006). A multichannel femtoampere-sensitivity potentiostat array for biosensing applications. IEEE Trans. Circuits Syst. I Regul. Pap..

[9] Ayers S., Gillis K.D., Lindau M., Minch B.A. (2007). Design of a CMOS potentiostat circuit for electrochemical detector arrays. IEEE Trans. Circuits Syst. I Regul. Pap..

[10] Bozorgzadeh B., Schuweiler D.R., Bobak M.J., Garris P.A., Mohseni P. (2016). Neurochemostat: A Neural Interface SoC With Integrated Chemometrics for Closed-Loop Regulation of Brain Dopamine. IEEE Trans. Biomed. Circuits Syst..

[11] Kara A., Reitz A., Mathault J., Mehou-Loko S., Amirdehi M.A., Miled A., Greener J. (2016). Electrochemical imaging for microfluidics: A full-system approach. Lab Chip.

[12] Hasan S.R. (2007). Stability analysis and novel compensation of a CMOS current-feedback potentiostat circuit for electrochemical sensors. IEEE Sens. J..

[13] Ahmadi M.M., Jullien G.A. (2009). Current-mirror-based potentiostats for three-electrode amperometric electrochemical sensors. IEEE Trans. Circuits Syst. I Regul. Pap..

[14] Martin S.M., Gebara F.H., Strong T.D., Brown R.B. (2009). A fully differential potentiostat. IEEE Sens. J..

[15] Rodriguez-Villegas E. (2009). A low-power wide-range IV converter for amperometric sensing applications. IEEE Trans. Biomed. Circuits Syst..

[16] Yang C., Huang Y., Hassler B.L., Worden R.M., Mason A.J. (2009). Amperometric electrochemical microsystem for a miniaturized protein biosensor array. IEEE Trans. Biomed. Circuits Syst..

[17] Ahmadi M.M., Jullien G.A. A very low power CMOS potentiostat for bioimplantable applications. Proceedings of the IEEE Fifth International Workshop on System-on-Chip for Real-Time Applications (IWSOC’05).

[18] Stanacevic M., Murari K., Rege A., Cauwenberghs G., Thakor N.V. (2007). VLSI potentiostat array with oversampling gain modulation for wide-range neurotransmitter sensing. IEEE Trans. Biomed. Circuits Syst..

[19] Genov R., Stanacevic M., Naware M., Cauwenberghs G., Thakor N. (2006). 16-channel integrated potentiostat for distributed neurochemical sensing. IEEE Trans. Circuits Syst. I Regul. Pap..

[20] Schienle M., Paulus C., Frey A., Hofmann F., Holzapfl B., Schindler-Bauer P., Thewes R. (2004). A fully electronic DNA sensor with 128 positions and in-pixel A/D conversion. IEEE J. Solid-State Circuits.

[21] Stanacevic M., Murari K., Cauwenberghs G., Thakor N. 16-channel wide-range VLSI potentiostat array. Proceedings of the 2004 IEEE International Workshop on Biomedical Circuits and Systems.

[22] Zoski C.G. (2006). Handbook of Electrochemistry.

[23] Nazari M.H., Genov R. A fully differential CMOS potentiostat. Proceedings of the 2009 IEEE International Symposium on Circuits and Systems.

[24] Wang W.S., Kuo W.T., Huang H.Y., Luo C.H. (2010). Wide dynamic range CMOS potentiostat for amperometric chemical sensor. Sensors.

[25] Sackinger E., Guggenbuhl W. (1987). A versatile building block: The CMOS differential difference amplifier. IEEE J. Solid-State Circuits.

[26] Mahmoud S.A., Soliman A.M. (1998). The differential difference operational floating amplifier: A new block for analog signal processing in MOS technology. IEEE Trans. Circuits Syst. II Analog Digit. Signal Process..

[27] Soltan A., Soliman A.M. (2009). A CMOS differential difference operational mirrored amplifier. AEU-Int. J. Electron. Commun..

[28] Alzaher H., Ismail M. (2001). A CMOS fully balanced differential difference amplifier and its applications. IEEE Trans. Circuits Syst. II Analog Digit. Signal Process..

[29] Harrison R.R. A versatile integrated circuit for the acquisition of biopotentials. Proceedings of the 2007 IEEE Custom Integrated Circuits Conference.

[30] Baker R.J. (2008). CMOS: Circuit Design, Layout, and Simulation.

[31] Gosselin B., Sawan M., Chapman C.A. (2007). A low-power integrated bioamplifier with active low-frequency suppression. IEEE Trans. Biomed. Circuits Syst..

[32] Prakash S.B., Abshire P., Urdaneta M., Christophersen M., Smela E. A CMOS potentiostat for control of integrated MEMS actuators. Proceedings of the 2006 IEEE International Symposium on Circuits and Systems (ISCAS 2006).

[33] Al Mamun K.A., McFarlane N. A CMOS potentiostatic glucose monitoring system for VACNF amperometric biosensors. Proceedings of the 2015 IEEE International Symposium on Circuits and Systems (ISCAS).

[34] Li H., Liu X., Li L., Mu X., Genov R., Mason A.J. (2017). CMOS Electrochemical Instrumentation for Biosensor Microsystems: A Review. Sensors.

[35] Kuno T., Niitsu K., Nakazato K. (2014). Amperometric electrochemical sensor array for on-chip simultaneous imaging. Jpn. J. Appl. Phys..

[36] Niitsu K., Ota S., Gamo K., Kondo H., Hori M., Nakazato K. (2015). Development of Microelectrode Arrays Using Electroless Plating for CMOS-Based Direct Counting of Bacterial and HeLa Cells. IEEE Trans. Biomed. Circuits Syst..

